# Assessing rural household’s food groups-and-sources and dietary diversity pattern in Malaita Province

**DOI:** 10.1038/s41598-023-39124-3

**Published:** 2023-08-15

**Authors:** Zina Bird, Viliamu Iese, Helene Jacot Des Combes, Bradley Alungo, Morgan Wairiu

**Affiliations:** 1 Pacific Centre for Environment and Sustainable Development (PaCE-SD), The University of the South Pacific, Suva, Fiji; 2 National Disaster Management Office, Ajeltake, Marshall Islands; 3 Islands Knowledge Institute, Honiara, Solomon Islands

**Keywords:** Climate change, Climate sciences, Health care

## Abstract

Access to and availability of various food sources is not an issue in rural communities. However, there is no guarantee that households are not affected by nutritional inadequacy, which is still a problem in most underdeveloped nations. A mixed-methods study was conducted to determine the HDDS through the snowballing method for the last 12 months’ food groups-and-sources and the 48-h diet recall. Ninety-eight households in two rural communities surrounding Sikwafta (zone one) and Malu’u (zone two) participated in the interview between April and May 2019. The results were then analyzed through SPSS and QDA MINER. The aim is to analyze the household dietary patterns of the communities between the two zones. The study also hypothesizes that the dietary pattern of households has not changed. Results showed that a total of nine food groups were consumed: grain, white roots, tubers, plantains, oils/fats (95.90%), condiments (83.70%), and meat/poultry/sea foods (77.60%). The majority of the food consumed comes from the gardens, followed by other food sources, which make up the average HDDS of five. A correlation analysis found a statistically significant relationship between HDDS and total meal (0.504*), with no relationships between demographics. When comparing 48-h food sources-and-groups to the previous year, there was little to no difference in accessibility and availability. This implies that the majority of households are likely to achieve their nutritional needs within the parameters of their dietary trend. Even though households have adequate access to food, there is a significant need to improve their nutritional needs, even if they consume the average amount of the required food groups.

## Introduction

Dietary patterns are extremely important to human health. According to several studies^1–4^, an unbalanced or unhealthy diet has continued to raise health concerns due to dietary and nutrient intake deficiency, contributing to the global burden of health problems. Globally, the most common health complications related to diets are cardiovascular diseases (CVD) or non-communicable diseases (NCDs).

The change in food preferences has also resulted in progressively inadequate nutritious diets, resulting in over or undernutrition. For instance, in the last few decades, the Asia–Pacific region's food consumption patterns have shifted dramatically^5^. Since the 1980s, as mentioned in^6–8^, there has been a growing shift in healthy dietary patterns from indigenous traditional diets centered on tubers, legumes, fresh fish and meat, green leafy vegetables, nuts, whole grains, and fruit to a more westernized diet centered on saturated fatty and processed meats, refined rice, oils, and sweets. This shows an increasing reliance on ultra-processed diets high in salt, sugar, and saturated fats.

Malnutrition is a problem in the Asia–Pacific region. Like many countries going through the diet transition, lack of nutritional energy and protein and micronutrient deficiencies have led to overweight and obesity in the region, even within the same household and sometimes within the same person because of the diet transition^5^. NCDs are responsible for 71.00% of global mortality and disability, with the majority of cases occurring in low- and-middle-income countries^9^. Within the Pacific Island Countries and Territories (PICTs) themselves, NCDs are responsible for 80.00% of deaths and 75.00% of the obese population^9^. Between 2010 and 2014, there was an increase in overweight adults from 29.30% to 33.00% in the region, with 5.20% of children under the age of five years^10^. Because of the rising prevalence of malnutrition due to diet transitions, Sustainable Development Goal two intends to eliminate all types of malnutrition through the use of four nutritional indicators: stunting and wasting in children under the age of five; anemia in women of reproductive age (15–49 years), and childhood obesity^11,12^.

The Solomon Islands is one of the 13 countries in the Small Islands Developing States (SIDS)^13^, is no stranger to malnutrition and health problems caused by the consumption of insufficient essential minerals and vitamins in their diet. This is due to the high consumption of large quantities of cheap calories in the form of saturated fat and sugary foods, leading to NCDs ^14^. According to the Global Nutrition Report^15^, the proportion of obese women (30.40%) in the Solomon Islands is higher than that of men (21.00%). However, it is lower compared to other Pacific regions (31.70% female and 30.50% male). Much of this problem is influenced by lifestyle factors such as food preferences, economic access, or physical activity^16,17^. It implies that households are not immune to hunger and associated health challenges because of limited economic accessibility to nutritious food, which results in poor nutrition (malnutrition)^16,18^.

According to food consumption data obtained in the 2012/13 Household Income and Expenditure Survey (HIES)^17^, the population in the Solomon Islands consumed around 2640 kcal per day, indicating high access to excessive amounts of energy-containing foods in their diet while one in ten people was undernourished, equivalent to approximately 56 000 individuals being hungry^17^. This may still indicate that the Solomon Islands population is lower in comparison to the global scale, with approximately 21.00% of the African population, 9.00% in Asia, and 9.10% in Latin America and the Caribbean affected by hunger or malnutrition in 2020^19^. However, inequality of calories gained from diets remains an issue in the Solomon Islands, where high-income households have greater access to foods with higher calories than low-income households, particularly those at the bottom of the distribution, where 20.00% of the total expenditure is spent on food^17^. The inequality of calories gained was also influenced by the socio-demographic characteristics of households. Female-headed households, for example, frequently consume more than male-headed households^17^.

Looking at the SIDS population, there is an uneven distribution in the dietary pattern. According to Haynes et al.^13^ and Peng et al.^20^, the majority of the populations in the SIDS are experiencing an alarming increase in poor nutritional diets, which is affecting their health and contributing to over- or undernutrition. In the Solomon Islands, the diet is relatively homogeneous and not diverse. At the national level, it is balanced in terms of the contributions of proteins, fats, and carbohydrates to the total dietary energy consumed. Although reports show a balance in diet consumption, only 17.00% of Solomon Islanders have sufficient access to these proteins, fats, and carbohydrates to meet all recommended dietary guidelines.

Several studies^2,13,21,22^ reported how consuming too much or too little of one food group might trigger health problems resulting in malnutrition. Furthermore, the issues of malnutrition, NCDs, and being overweight still exist despite the efforts to eradicate hunger^23,24^. While this issue exists in many rural communities, limited studies have been conducted on the dietary patterns of households, which is important to the overall understanding of food and nutritional security, economic accessibility, and its health implications at the sub-national level.

**Figure 1:**
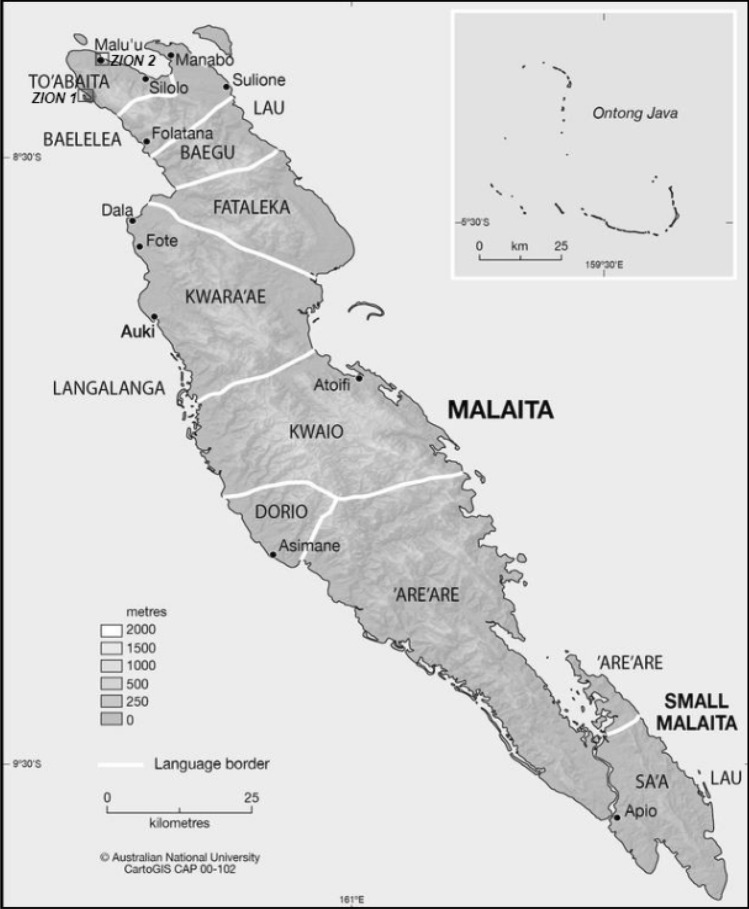
Locations of the study sites divided into two zones in the To'abaita region – Adopted an modified from CartoGIS Services, College of Asia and the Pacific, The Australian National University, under a Creative Common lience ((License: CC BY-SA 4.0 https://creativecommons.org/licenses/by-sa/4.0/).

Therefore, this study aimed to analyze the household dietary patterns of the communities surrounding zone one and zone two in North Malaita over two days (48 h) using the 24-h dietary recall (Fig. 1). The study also hypothesizes that the dietary pattern of the households has not changed in the past 12 months (2018) for the two selected rural communities.

## Methods

### Identifying participants

#### Study Population and sampling

**Figure 2:**
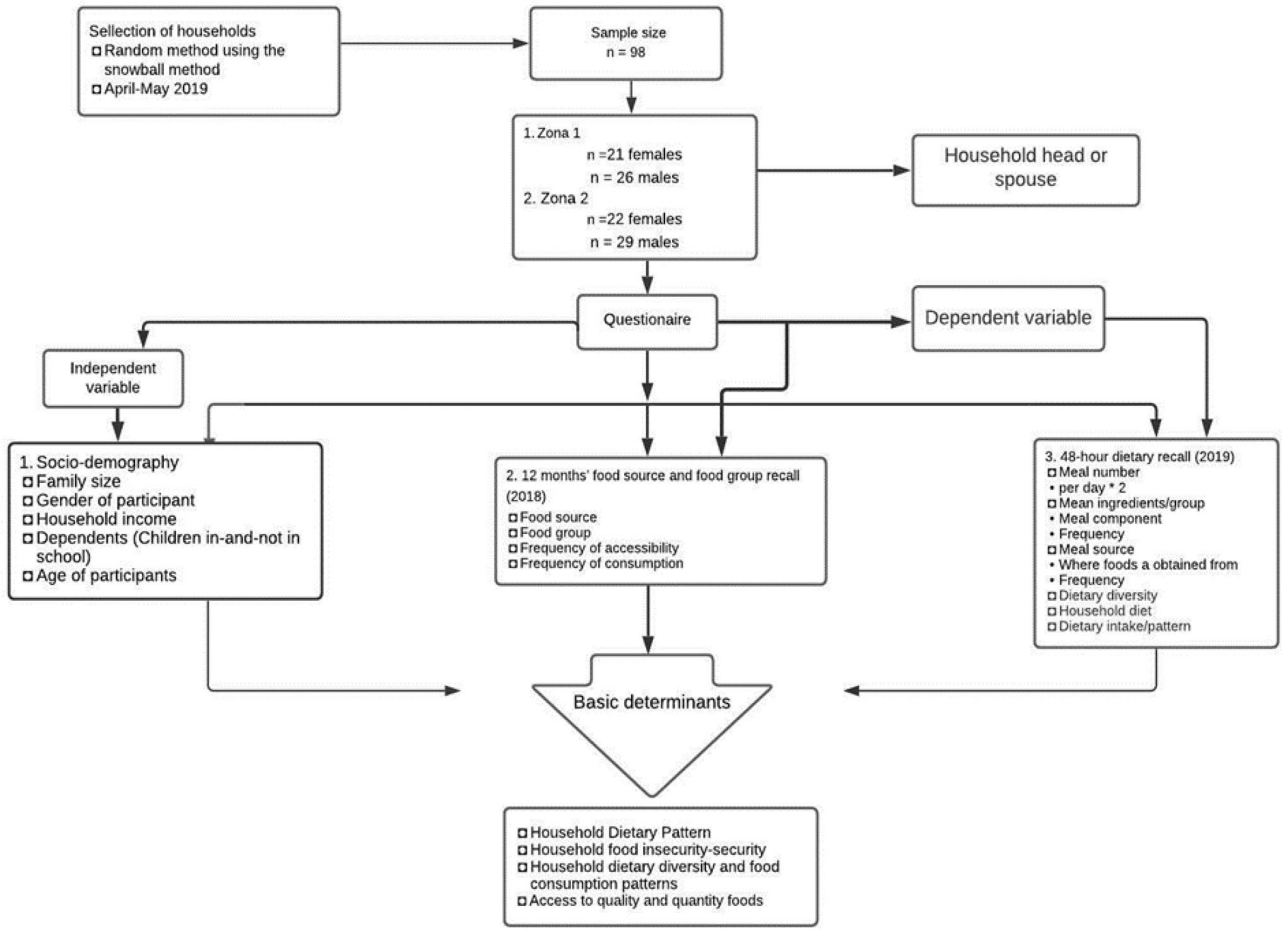
Flow chart of the study variables, determines and questionnaire structure on household dietary pattern.

Communities in the To’abaita region of Malaita were the targeted study population. The communities were separated into two zones. Zone one involves communities surrounding Sikwafata, and zone two involves communities within the Malu’u boundary. To have an equal representation of the households in each zone, the target for each zone is 50 households. However, due to unforeseen circumstances when dealing with a two-day diet recall (HDDS), some of the households were unavailable during the second day of the survey, and their survey questionnaires had to be dismissed to avoid inconsistencies. Considering the unavailability of some of the households, each zones has an extra one sample size. As a result, 47 households were interviewed in zone one, and 51 households (Fig. 2) were interviewed in zone two, for a total of 98 households. The eligibility criteria include heads of households or their spouses who were local farmers and responsible for preparing household meals (Fig. 2). This will be covered in more detail under the 48-h diet recall.

To ensure random sampling, the snowball approach was chosen as a means of collecting data. It was chosen because of its nature as one of the qualitative research methods in which the researcher contacts informants using prior informants' information, who then recommend to the researcher the next possible interviewee, and so on^27^. Additionally, since the two selected zones (study sites) are not clustered (distributed from the shoreline to the mountains), snowballing is a better method option and more effective for data collection. This method was chosen to limit households selected (if using random sampling) who were not available during the time of the survey. Thus, since snowball sampling is a non-probability sampling approach (the method of selecting participants from a population using a subjective method or the first informants contacted), it is a quick, convenient, time-saving, and simple way to collect data that does not necessitate a full survey frame. For instance, through a random approach. Thus, the researcher contacted informants based on the information provided.

### Data collection

The data were collected between the months of April and May of 2019 specifically targeting the local farmer's availability. Participants were interviewed based on their availability and were informed of the study's ethical context, which did not intend to harm their households in any way. With that, an informed consent was obtained from all the participants included in the study. Before the study was performed, we ensured that it followed the ethics of the University of the South Pacific research, for which the proposal to conduct the study was approved by the “*University of the South Pacific Research Ethics Committee*”. All the methods and protocols were adhered and strictly based on the FAO guidelines for measuring household and individual dietary diversity: minimum Dietary Diversity-Women (MDD-W), WHO Food safety, and CFaH toolkit guidelines to set up this research methods. These methods are further elaborated according to their respected headings. 

The survey was also specifically developed to interview the person who prepares household meals as criteria for inclusion or exclusion, as shown in Fig. 2. Figure 2 represents the research design, or a simple diagram of the methodology. The interview schedule consisted of questions on the demographic characteristics, food groups-and-sources in the last 12 months, and dietary recall for two days using the 24-h diet recall equivalent to the 48-h diet recall administered during the time. Each of the data collection methods is discussed further under their respective allocated headings:

1: *12 months’ food groups-and-sources recall*,

2: *Dietary Pattern—48-h Diet recal*l., and.

3: *Calculating Food Consumption Score (FCS).*

To calculate the correlation for the study, demographic characteristics will be used to investigate any relationships between the demographic, HDDS, and FCS.

During the interview, pidgin was used as a tool for verbal communication before being translated back into English for transcription and analysis. The sample size was specifically selected because of the duration of the study. If there is a large sample size, covering the sample size within a short duration will take longer considering that the communities within the zones are scattered or dispersed. With the site’s geography in mind, the total sample size was the targeted sample size selected for the research.

The interviews were adopted from the Community Food and Health Project quantitative (CFaH) toolkit^28^ and modified for the study. The modified toolkit was based on the pre-existing instruments, which were co-designed by researchers from the Pacific, Caribbean, and United Kingdom^13^ to gather the study’s desired data. The data collected comprised the socio-demographic characteristics, 12 months’ food groups-and-sources recall, and dietary patterns involving 48-h diet recalls and household dietary diversity.

#### 12 months' food groups-and-sources recall

The 12-month's food groups-and-sources recall as a reference point was developed to access the previous food groups-and-sources obtained by the 98 households through the targeted participants in the two selected zones. An equal number of household’s sample size was targeted based on the site's geography. This provided a useful overview of the types of food groups-and-sources obtained within the previous 12 months, considering that some food categories are only available and accessible on a seasonal basis.

The interview involves collecting the frequencies and total food groups the household head or spouse recalled having access to, consuming, and the sources from which they obtained the food prepared for family meals. The food source data were collected based on the level of accessibility: daily, two–three times a week, weekly, monthly, infrequently, never obtained, and fortnightly. The 12-monthly recall was designed similarly to the 48-h diet recall method with 13 food groups; however, only food groups-and-sources were asked based on their recollection of the previous 12 months in their households. The food groups in this study were adapted and modified from the FAO’s Minimum Dietary Diversity-Women (MDD-W) questionnaire, which resulted in the final number of food groups of 13 (Table 1).

**Table 1:** Main food groups.

Food group number	Food group
1	Grain, white roots and tubers, and plankton
2	Pulse
3	Nuts and seeds
4	Dairy products
5	Meat, poultry, sea food, insects
6	Egg
7	Dark green leafy vegetables
8	Other vitamin A-rich fruits, vegetables and roots and tubers
9	Other vegetables
10	Other fruits
11	Condiment and spice
12	Sugary products (beverages/tea/sweets)
13	Oil/fats
Optional food group*	
Savory and fried snacks	
Alcohol	
Other non-sugar/alcohol beverages	

*The optional food group is only for transparent recollection, primarily to provide a space for data collectors to mark these foods and beverages to prevent falsely categorizing them under the main 13 food groups when collecting data with the open recall method over the previous 12 months.

Some of the 13 food groups were subdivided further to allow for the collection of data on additional foods. Some of the food groups for the DD were combined, while others were separated as individual food groups. For example, vitamin A-rich foods were separated from other fruits and vegetables, and a combination of high-carbohydrate or starchy food groups was divided between white and highly vitamin-rich groups. Insects were included under the meat, poultry, and seafood food groups as they were considered protein substitutes and are mostly consumed fresh in the context of the study sites. Because a wide variety of insects and other small protein foods are also highly nutritious, insects are also nutrient dense and can provide protein, fatty acids, and micronutrients when consumed as part of a meal; thus, they are categorized under food group five (Table 1). Although tinned meat/fish and insects have different weights according to the food consumption score proxy, we combined them under fresh meat, poultry, and seafood as protein intake as indicated by the WDD-W food groups.

Although it is critical to consider the availability of food, including seasonal food items under specific food groups is a good indicator for family behavior around food consumption and dietary pattern, in this study. The data from previous surveys, documentation of past eating habits, and dietary recall will be used as an approache to address behavior around food consumption over a year to understand the dietary behavior of households.

#### Assessing Dietary Pattern—48-h Diet recall

One of the key focus is to assess the dietary pattern of the households as measured through the following meal characteristics:

### Dependent variables and their measurement

***Meal numbers per day:*** Number of meals consumed by a household in the 48-h recall (2 day period). Foods consumed outside of households by individuals were not included.

***Meal ingredients:*** Meal ingredients are the meal items that comprise a meal, or what the meal is made up of. Two different indicators were used to assess the meal's ingredients. These were the HDDS and FCS developed by the Food and Agriculture Organization of the United Nations (FAO) as indicators to measure household food security.

***Meal source:*** The meal source is the location from which the foods or ingredients in a meal are obtained. The meal source is determined by the number of sources of foods gathered and prepared by the household in the past 48-h. The meal source is the place from which the foods or ingredients in a meal are obtained. The meal source is determined by the number of sources of foods gathered and prepared by the household in the past 48-h.

Using dietary patterns to assess dietary quality provides a useful linkage emphasizing either high-quality or low-quality diets that include numerous food groups and beverages known as DD. It determines if a diet is of high or low quality. Thus, DD, which is defined as a “qualitative measure of food consumption that reflects household access to a variety of foods and is also a proxy for the nutrient adequacy of individuals’ diets”^25^, is also important to consider when assessing dietary patterns.

Aside from other methods used worldwide to measure human nutrition, such as the anthropometric measures and measures of calorie intake used by nutritional experts, the HDDS and FCS were designed for non-nutritional researchers who have some technical expertise to assess the status of an individual or household food security. These two indicators have been validated in other countries as micronutrient measures associated with individual and household nutritional quality. Belgium, Mali, Bangladesh, and Papua New Guinea were among some of the countries where HDDS and FCS were validated^30–33^. Thus, the HDDS and FCS have been demonstrated to be good indicators for assessing household dietary patterns and food security.

The HDDS is a widely used tool for assessing dietary quality efficiently, especially for monitoring and evaluating nutrition and food security. It also has accessibility features^26^. The scores collected through questionnaires indicate the food frequency, dietary quality, and nutrient intake of every meal. Therefore, HDDS is attributed to nutrient intake adequacy and nutritional requirements for different population groups. 

The household diet was designed to explore the variance in the variety of foods consumed by households based on diet recalls. This method was widely recognized and used by many studies globally, such as^33–35^, and ^36^, to determine the dietary pattern and subsequently the nutritional status of households. This proxy reflects the household's economic status as well as nutritional sufficiency in a diet^25^. Thus, for this study, the current dietary status or diet recall was conducted using the basic HDDS, with 13 food groups (Table 1). A 24-h diet recall was administered over a two-day reference period, giving a total of 48-h of recall to avoid errors in recalling over a longer period for the same 98 households. The households were visited every 24-h to record their previous meals. The design assesses the level at which a household can afford a wide range of diets, indicating whether they are capable of accessing a greater quantity of food and beverages and at what frequency^37,38^. According to the HDDS design, only the spouse or person responsible for meal preparation was interviewed.

#### Calculating HDDS

To obtain the HDDS, the required data are collected through the 48-h dietary recall, which records every food ingredient consumed within the household in every meal. The same 13 food categories were employed in the last 12 months. As a result, the HDDS is measured from scores 0–13 representing the food groups. Though the average of 13 food groups is six-point five or seven, for this study, we will be comparing this average with the standard average of five food groups according to^37^ as the recommended intake. On average, those who consume more than five food groups are more likely to satisfy micronutrient demands, meet the necessary food group consumption, or have good economic accessibility than those who consume fewer food groups^39^.

The food frequency data gathered during the 48-h recall is used to evaluate food sources and dietary patterns. The score was assigned based on the food categories ingested in the previous 48-h. The total number of food groups can be calculated as follows: households with higher scores are more likely to be nutritionally secure. This would also imply that they have sufficient purchasing power to obtain high-quality foods and other condiments based on their dietary preferences. Households with lower scores, on the other hand, are nutritionally insecure, have limited purchasing power, and can only afford locally produced staple foods. To calculate the HDDS, the following equation is used:

$$ \begin{gathered} {\text{HDDS }}\left( {{\text{score range from }}{\text{0-13 per day}}} \right) \, = {\text{ Sum }}\left( {{\text{a}} + {\text{b}} + {\text{c}} + {\text{d}} + {\text{e}} + {\text{f}} + {\text{g}} + {\text{h}} + {\text{i}} + {\text{j}} + {\text{k}} + {\text{l}} + {\text{m}}} \right), \hfill \\ {\text{Average HDDS }} = \, \left( {{\text{a}} + {\text{b}} + {\text{c}} + {\text{d}} + {\text{e}} + {\text{f}} + {\text{g}} + {\text{h}} + {\text{i}} + {\text{j}} + {\text{k}} + {\text{l}} + {\text{m}}} \right) \, /{\text{ Number of Households}} \hfill \\ \end{gathered} $$

Note that although condiments (fresh, dried, and processed) and oil/fat consumption often relate to food preparation in moderate quantities, they are included in all meal preparations and are common ingredients.

#### Calculating FCS

A 48-h dietary recall survey was also used to calculate the FCS over the two days, or in other words, obtain it through the diet recall. Instead of the standard seven-day dietary recall, a two-day recall was implemented to observe the dietary patterns. Primarily, this is due to the time frame and location of the homes in the two different zones. The FCS measures food quality. To obtain the FCS, the person responsible for the food preparation is interviewed and listed the foods prepared throughout the 48-h recall. The total frequency of the foods consumed in the two days is then multiplied by the allocated weights of the food groups (Table 6). Thus, the sum gives the FCS.

$$ \begin{aligned} {\text{FCS }} & = {\text{ Total Food group }}\left( {\text{two days}} \right) \, \times {\text{ allocated weight}} \\ & = \, \left( {{\text{Total Cereal}}/{\text{tuber }} \times {\text{ weight}}} \right) \, + \, \left( {{\text{total Pulses }} \times {\text{ weight}}} \right) \, + \, \left( {{\text{total Vegetables }} \times {\text{ weight}}} \right) \, + \, \left( {{\text{total Fruits }} \times {\text{ weight}}} \right) \, + \, \left( {{\text{total Meat}}/{\text{fish}}/{\text{insect }} \times {\text{ weight}}} \right) \\ & \quad + \, \left( {{\text{total milk }} \times {\text{ weight}}} \right) \, + \, \left( {{\text{total Sugar }} \times {\text{ weight}}} \right) \, + \, \left( {{\text{total Oil }} \times {\text{ weight}}} \right) \, + \, \left( {{\text{total Condiments }} \times {\text{ weight}}} \right) \, + \, \left( {{\text{total Tinned fish}}/{\text{meat }} \times {\text{ weight}}} \right) \\ \end{aligned} $$

### Independent variables and their measurement

#### Measuring demographic characteristic

In this study, the socio-demographic characteristics were treated as the independent variable. The selected socio-demographics are as follows: Note that gender will not be included in the correlation analysis involving the five socioeconomic variables because the gender characteristic is treated as nominal data that cannot be ranked; thus, it was not assigned a number to conduct correlation.

I. Family size: total number of family members living in the same household
II. Dependent: total number of children in school and not in school,
III. Income: total income earned by the whole family
IV. Age: number of years of the participants, and
V. Gender of participant

These were chosen as a means to assess their role in the dietary patterns of the households. Note that the demographic data is based on existing data already published in^29^. It is also important to note that the age was focused only on the interviewee (spouse or household head). The household's income is based on the total number of members engaged in any form of income-generating activities that contribute to the household’s dietary pattern, with the assumption that the more working members there are, the greater their food purchasing power. The dependents were selected to identify how the food will or may be distributed according to children attending school or at home. These characteristics were chosen to assess how they can influence the dietary pattern and the food security status of the households.

#### ***Data analysis***

To obtain the statistical result of the data collected through the 48-h diet recall, 12 months of food source-and-group, correlation, and demographic characteristics, two statistical software packages were used in this study. The primary DD analysis was done through the use of the Statistical Package for the Social Sciences (SPSS). The data were imported from Excel into SPSS software for analysis of the two-day diet recall (48-h dietary recall), accessibility, source, and HDDS. The software was also used to generate the correlation between HDDS and the household socio-demographic to investigate relationships or whether one variable affects the other through the 1-tailed correlation analysis (**Correlation is significant at the 0.01 level and *Correlation is significant at the 0.05 level). The 12 months’ food groups-and-sources recall were analyzed using QDA MINER software, a software used for qualitative data. The qualitative questionnaire was imported into QDA MINER through Microsoft Word, where codes and categories were then assigned for the responses. Each of the given codes, also known as the codebook, displaying the tree structure, is placed under its assigned category. The categories are the nodes under which associated codes can be found and analyzed. Selected text segments from the transcripts are assigned to the codes, which are then grouped into categories (or main topics). The results generated from the two statistical software programs will be both descriptive and inferential statistics. The inferential data will be the correlation result to test the hypothesis if there is any significant relationship between the variables. These variables include the socio-demographics, HDDS and FCS.

The results from this analysis can be found and further discussed in the result and discussion sections. Note that the study has both descriptive and inferential components. All data are inferential except gender, food source, and food group (12 months).

## Results

### Demographic characteristics 

Presented in Table 2 are the results of the socio-demographic characteristics of the households, showing their mean and standard deviation. The average family size for the 98 households ranges between four-point nine and five-point on members, with an average age of 41.7 (42) and 50.3 (50) in zones one and two. However, in total, the average age is 46.2 years. The participants were classified into three age groups: youth (18–35), middle age (36–55), and old age (56+) (Table 2). According to the findings, 505 individuals are living within the 98 households (the sample size). A total of 212 out of the 505 family members are children, representing dependents, one of the key focus demographics. The remaining were youths and adults. Of the children under the age of five years, 84.00% attended school, while 16.00% remained at home. Households' weekly income ranges from USD 2–212 with an average of USD 48.8. The household’s income was calculated per household. All the households were involved in farming. Income was earned through the sale of farm and marine products (92.70%). Only 7.20% have a steady source of income. The selected demographic characteristics discussed above will be used to determine the dietary pattern of the households represented in the correlation section.

**Table 2:** Statistical results of the demographic Characteristics.

Demographic characterisitic	Mean	SD
Age	46.2	16.1
Youths (18–35)	25.9	10.2
Middle-age (36–55)	43.3	6.0
Old age (above 56)	60.1	20.1
Family size	5.1	2.4
*Dependents*		
In school	1.8	1.9
Not in school	0.3	0.6
Total	2.1	2.0
Income	USD 48.8	67.8

Nominal data		
Gender	Male	Female
	55	43

### Dietary pattern for households for the last 12 months

One hundred percent of the households’ diets consisted of mainly oils/fats, vegetables/crops, nuts/seeds (99.00%), condiments (96.90%), fruits (95.90%), sugar-sweetened beverages and legumes (94.80%), fresh meat (80.4%), cereal/grain (78.40%), savory food (64.90%), and sweet food or snacks (53.60%). Other food groups, such as insects, non-alcohol beverages, eggs, alcoholic beverages, pre-cooked food, and dairy products, were also important components of the diet, but were found to be less common. Furthermore, Table 1 shows the main 13 food groups further subdivided.

**Figure 3:**
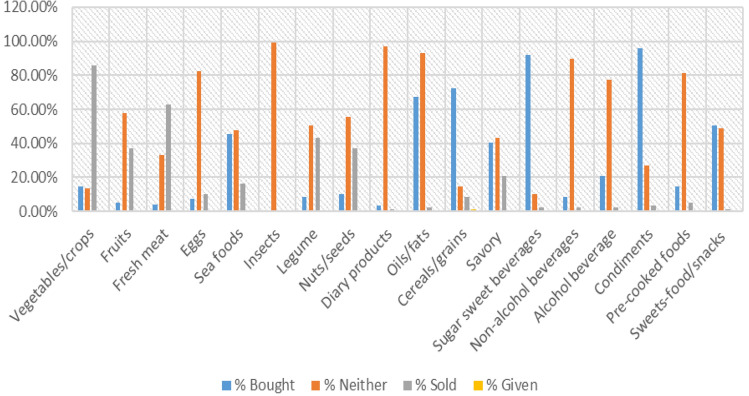
Trend of obtaining food ingredients in the last 12 months.

**Figure 4:**
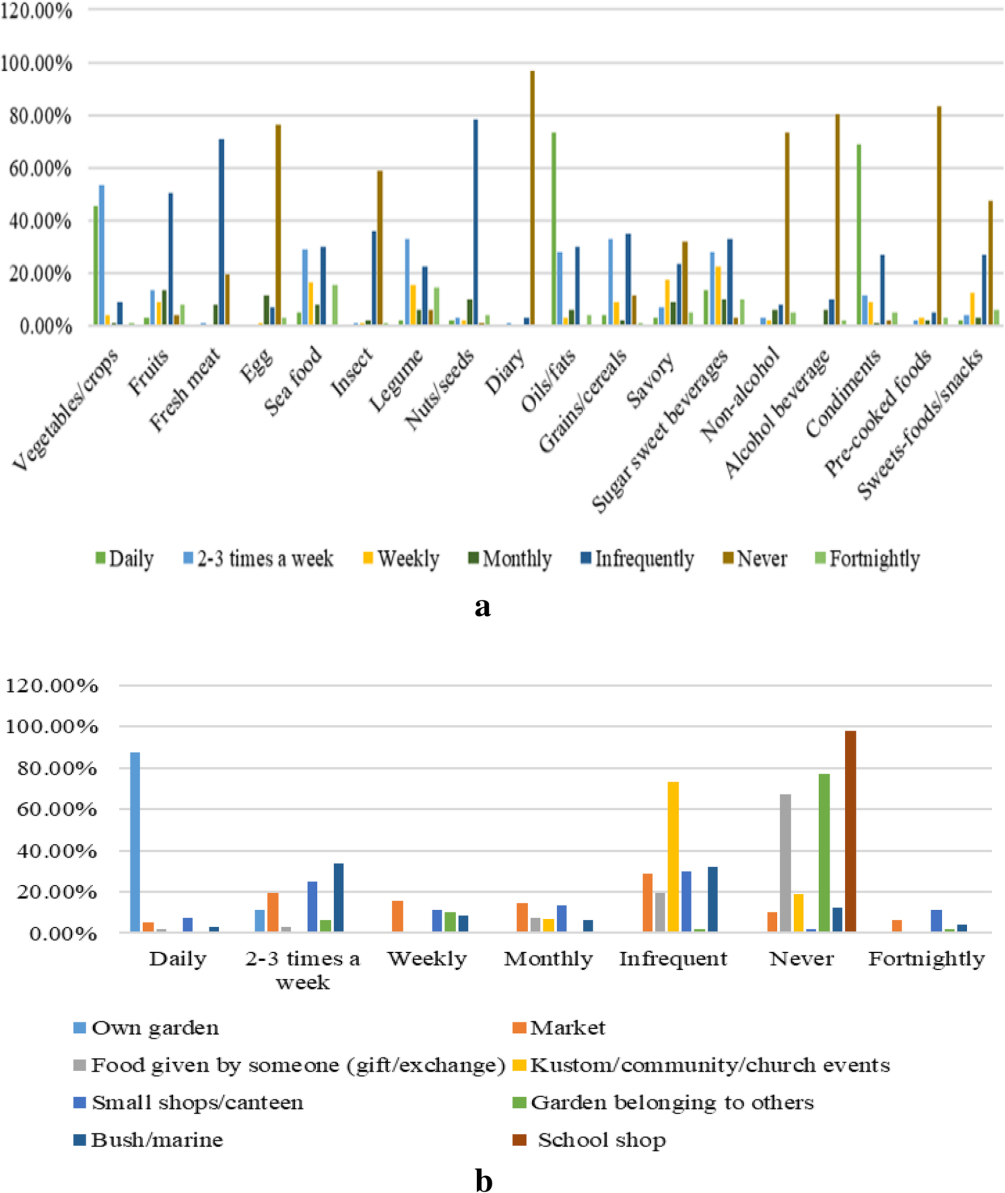
(**a**) percentage of food groups consumed (**b**) food source recalled in the past 12 months.

**Figure 5:**
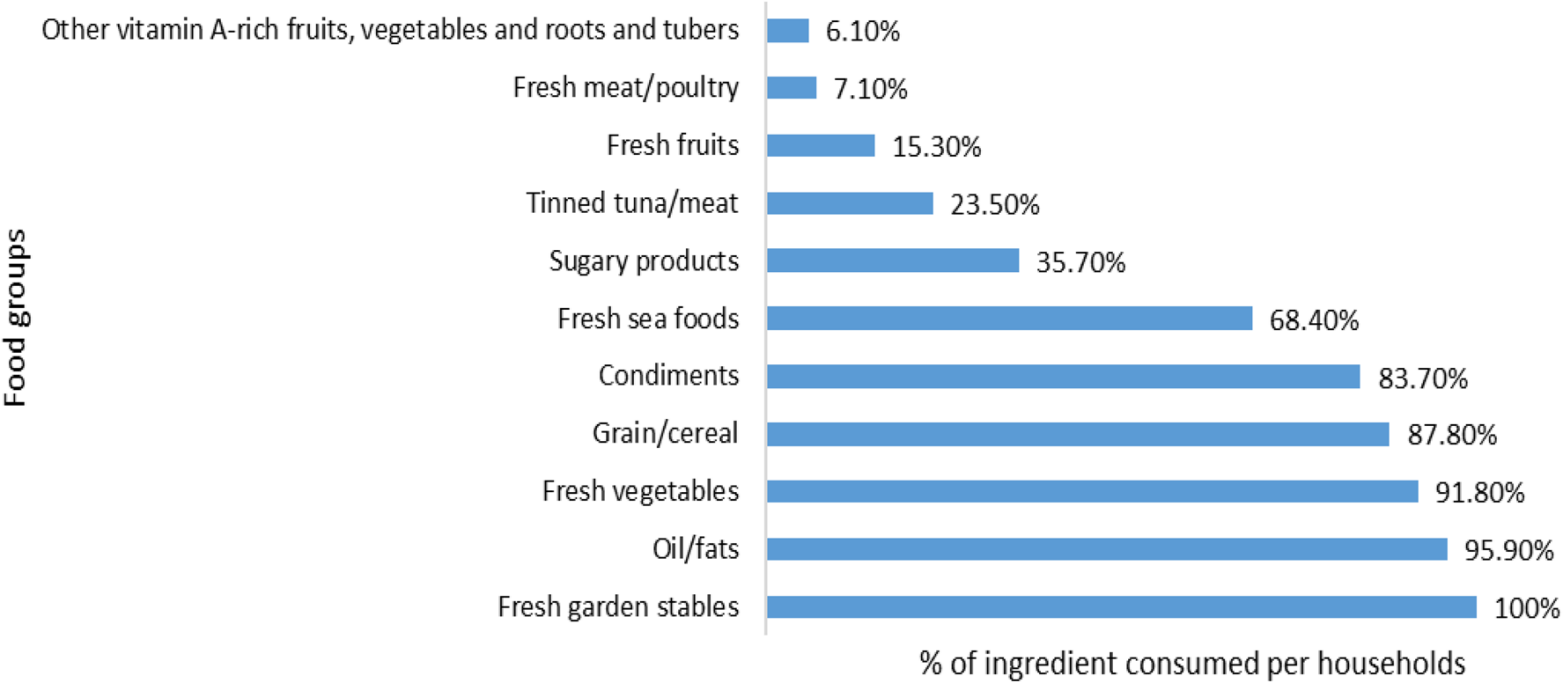
Main meal component within the 48-h period.

Taro (94.80%) and sweet potato (93.80%) were the two crops that predominated in the households’ diets. This is often supplemented by grains such as rice (68.0%). Proteins were largely obtained from sources such as pork (73.20%), shells (71.10%), and sugary products (example., sugary tea products) (78.40%). Vegetables such as dark green leafy vegetables (80.40%), beans (93.80%), tomato and salt (96.90%), coconut milk (or cream) (97.90%), ngali nut, and fruits such as pineapple were also found as components of households’ diets. The individual food items within the food groups are illustrated in Fig. 4a,b and 5, while Fig. 3 presents the percentages of which foods are commonly bought, sold, or given by other families, either by indicating they were gathered from their gardens, bush, marine, or both.

### Access to foods and their sources 

The results, as shown in Fig. 4a, reveal how frequently households obtained various food items throughout the past year. The result (Fig. 4a) of the food items is not classified specifically according to the food groups as participants were asked to recall the items on the list and the frequency with which they were obtained to gather their common staple food items. Nonetheless, households did not have an issue accessing certain food groups. For example, our study showed households have high access to oils/fats and condiments daily. For other food groups, households only access them at certain times during the week. Slightly more than half of the households (59.00%) access vegetables two to three times a week. The other 44.00% of the household accesses vegetables either daily, infrequently, or never. These accessibility features are presented in Fig. 4a. We also found that certain food groups were accessed on certain occasions. This largely depends on their availability and affordability. For instance, food groups such as fruits, fresh meat, seafood, legumes, nuts/seeds, grain/cereals, sugar-sweetened beverages, and sugary foods/snacks.

Results revealed that households obtained food from their gardens, markets, small shops/school shops, bush/sea, the market, cultural/church/community events, ceremonies, livestock farms, and family and friends (Fig. 4b). Results (Fig. 4a) show that 100% of the local fresh produce (roots and tubers, vegetables, and fruits) is predominantly sourced from their gardens.

### The 48-h household dietary pattern in early 2019

#### Meal frequency 

On day one of the diet recall surveys, households in both sites reported an average of three meals. That is, households had breakfast, lunch, and dinner. On day two, households in zone one reported having an average of two meals per day (that is, only breakfast and dinner), while in zone two they had an average of three meals. Overall, for the last 48 h, households in both sites consumed an average of three meals per day (Table 3).

**Table 3:** Combined meal frequency for D1, D2 and HDDS for 48-h. Note, 0 indicates skipped, cannot recall or the interviewed person as not at home on the day of interest.

**Variable**	Day	Meals	Percentage	Mean
*Meal Frequency*				
Meal per days	1	1	1.00	2.6 (3)
		2	29.60	
		3	69.40	
	2	0	9.20	2.4
		2	23.50	
		3	67.30	

HDDS Category	**Frequency**		**Percentage**	**Mean**
2–3	9		9.10	
4–5	45		45.90	
6–7	42		42.80	5
8	2		2.00	

#### Most common meal ingredients within the 48-h recall

Presented in Fig. 5, the most common food category from the least to the most consumed is broken down from the six food groups identified. Out of the 13 food groups examined in this study, white roots, tubers, and plankton; meat, poultry, and seafood; vegetables; fruits; oils/fats; sugary products; condiments; and grain/cereal were common ingredients in households’ meals within the 48-h reference period. Among the white roots and tubers, sweet potatoes were the most consumed carbohydrate crop. Pork, chicken, fish, crabs, fish, octopus, squid, and tinned meat are common sources of protein in household meals. Vegetables consumed were mostly dark green leafy vegetables, such as slippery cabbage. Flour and rice were the two grain/cereal food groups bought and consumed; however, rice has the highest consumption rate among participants within the last 48-h. Condiments such as salt were also added to meals, though in small quantities. Sugary goods, in particular, sugar-sweet beverages such as tea products, coffee, milo, and sugar were mostly consumed during breakfast and sometimes during dinner.

**Figure 6:**
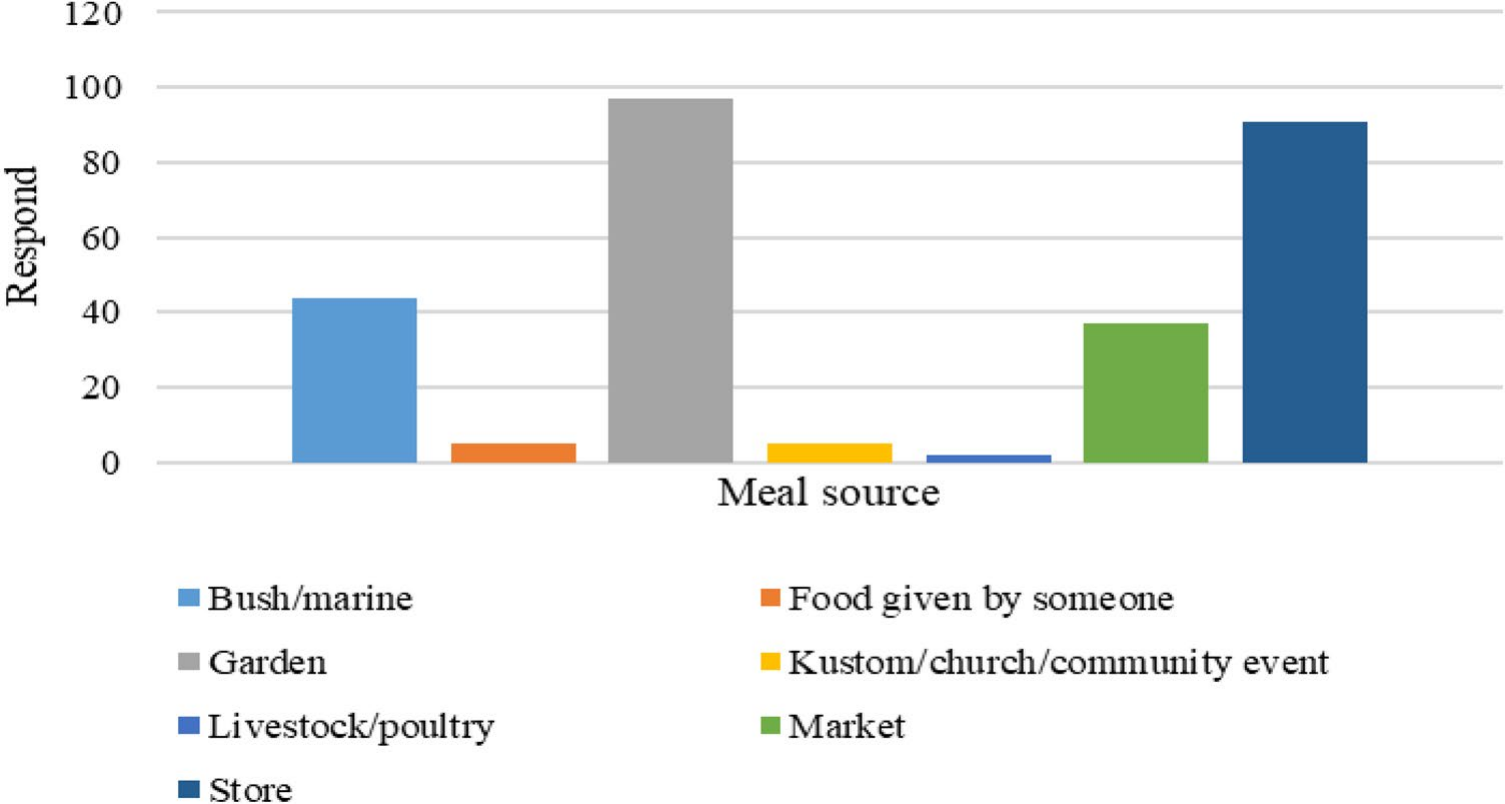
Food source for the 48-h diet recall.

Seven food sources have been identified (Fig. 6). The majority of households obtain their food from their gardens, followed by the store, the bush/marine, the market, kustom/church/community events, livestock farms, and food given by others (cooked or fresh). Despite having gardens, households also purchased fresh or cooked foods from the market for their households. Market purchases were made for four reasons: gardens were not ready for harvest, crops had not grown, they were damaged during extreme events such as cyclones and extended hot days, and going fishing was not possible for other reasons.

#### 48-h HDDS in 2019

The HDDS for both study sites ranged between two to eight food groups, with an average of five food groups. This indicated a consumption pattern of as few as two and as many as eight food groups per day. A typical diet, therefore, consists of five food groups, including grains, white roots and tubers, plantains (100%), oils/fats (95.90%), condiments (83.70%), dark green leafy vegetables (78.60%), and meat/poultry/seafood (77.60%). In a separate analysis for each study site, it was revealed that zone two consumed an average of six food groups, while zone one consumed an average of five food groups. These results suggested that 45.90% of the households in both sites were likely to satisfy their dietary needs; however, the probability of improving or declining diet is also high, while only a few (9.10%) were below the WHO requirement of four food groups per day or facing food and nutritional insecurity. The rest of the households (46.10%) have a substantial proportion to satisfy their dietary needs and have a stable diet.

Additionally, the FCS for the two days shows that condiments, grains, white roots, tubers, and plantains, and oil/fats had an average of two for the two days, indicating that they were consumed in all six meals. Dark green leafy vegetables, meat/poultry/sea foods, and sugary products showed an average of one, indicating a consumption pattern of one-two meals a day. Other fruits, vegetables, and vitamin-A-rich fruits and vegetables were the least consumed.

#### Correlation between household demographic, meals, and HDDS

A one-tailed correlation analysis was performed to assess the link between the 48-h dietary pattern of the households and selected demographics. The results (*p* = 0.05*, *p* = 0.01**) revealed both positive and negative significant relationships between the variables (Table 4). The age of participants had a negative correlation with total dependency and family size. This means the total number of dependents (− 0.313**) is high in households where the majority of household heads consist of youth and middle-aged groups and lower in households with older household heads. Age also has inversely significant effects (− 0.265**) on family size. That is, family size increases with decreasing age and vice versa.

**Table 4:** Correlation between dependent and independent variables.

Variables	Income (USD)	HDDS	Total dependent (Children)	Family size	Total meal (48-h)
Age of participant	–0.073	0.061	–0.313**	–0.265**	− 0.056
Income (USD)	–	0.114	0.103	0.149	0.174*
HDDS	–	–	0.034	–0.049	0.504**
Total dependent (Children)	–	–	–	0.591**	0.236**
Family size	–	–	–	–	0.215*

**. Correlation is significant at the 0.01 level (1-tailed).

*. Correlation is significant at the 0.05 level (1-tailed).

Results revealed a highly significant relationship between the 48-h meals and total dependency (0.236**), implying that the number of meals increases subsequently with an increasing number of dependents and vice versa. A similar result was observed for income (0.174*) and meal frequency. That is, households with high incomes will likely have a high number of meals and fewer meals among low-income households. Individually, income for zone one demonstrated a positive link with total meals for day two at 0.263* and family size at 0.275*, while not being reported in Table 4. In zone two, income was positively related to day one’s total meal (0.240*), day one's total food groups consumed (0.279*), and day two’s (0.321*) against HDDS. The study found a statistically significant (0.504*) connection between HDDS and the total meal. However, no correlations were found between HDDS, age, income, total dependency, and family size.

## Discussion

The purpose of this study was to look at the overall food groups-and-sources recalls for the past 12 months. The study also assessed households' dietary patterns and the components that make up their meals over a 48-h period. The results from both the 48-h recall and food groups-and-sources were used to investigate any change in the diet and food groups-and-sources pattern. The study presents the first community-based diet assessments measuring household economic accessibility to food for 48-h and previous years food groups-and-sources in the studied communities.

### The pattern between past and current food group-and-source

Consumption of locally grown fresh produce from the garden and marine/bush is an important source of healthy lifestyle patterns for the most rural population, including in the study population. From the results, their food groups-and-sources in the past year show a close pattern with their current 48-h DD. This indicates a typical consumption of food groups-and-sources for the households, as shown in the results of the analysis. For instance, in the past 12 months compared with the 48-h food groups-and-source, it shows a similar pattern, which indicates there has been little to no change in the food groups they consumed and the source from which these foods were obtained, especially fresh traditional foods. According to Campbell^40^, locally grown foods (or traditional foods) are embedded with cultural values of beneficence and kinship and will continue to provide resilience to the food systems for households or the rural population, which our study deemed the same. This was also seen in the types of locally fresh foods shown in the past 12 months and the 48-h diet recalls. This means that the rural populations are still depending on their locally grown food products and marine resources to meet their daily consumption requirements.

### Diet transition compared to other studies: how much is healthy?

The introduction of unhealthy processed diets or foods and beverages high in saturated fats, sugar, and salt has also influenced to some degree the change in consumption patterns in many households. This can be seen by the increase accessibility the sources at which foods are obtained from in the study such as the store-bought food items. This has led to a high increase in NCDs in the Pacific, leading to overweight and obesity, high blood pressure, high blood glucose, and high cholesterol levels, or, in other words, CVDs, chronic respiratory diseases, and diabetes because of excessive intakes, leading to the high death rate in the Western Pacific Region^10^. This also explains the different diet requirements for certain households, though this is not shown in the results relating to their health issues. This regional trend in consumption also explains the increase in the percentage of foods obtained from stores that were incorporated into the diets of the 98 households, especially condiments, sugary, and floury products. Similar to the findings of^41^. For example, diets in rural communities, as indicated in previous studies^2,3,17^, are more reliant on locally grown fresh produce (white roots and tubers, and plantains) and grains/cereal/wheat (rice and flour), for which our study found a similar pattern. This indicates that households still maintained their accessibility to their local foods.

However, the study also shows a shift or change in diet in their intake of food group one (Table 1). There have been records of rice becoming the most popular cereal/wheat product when compared to other cereal/wheat items, which also have a high percentage in current household diets. This transition was also supported by a study conducted by^17^. According to them, in the Solomon Islands, 19.00% to 23.90% of households consume rice in their diets. This implies that these two rural zones are undergoing a nutrition transition from a traditional diet to a mixture of modern diets at a slower rate compared to the populations^36,42^. It was also noticed from the current analysis that condiments, especially salt, have the highest intake. Tea products, sugar, and oils/fats especially coconut milk, were also recorded as high. Although these are common ingredients seen in the recalls, they can also affect health if households do not measure or control their daily intake. For example, although coconut milk is beneficial for health, it is calorie and fat dense. Meaning, a high-carbohydrate diet combined with excessive coconut milk consumption might also lead to weight gain and obesity.

For example^43^, reported that a fatty diet ranged from 244 g/d in the Solomon Islands to 989 g/d in Guam, while salt consumption ranged from 56 g/d in Kiribati to 103 g/d in Fiji. Only Guam reported absolute sugar consumption (473.3 g/d). Their findings show that sugar (50 g), salt (5 g), and oil (25–30 g) are consumed in excess of the recommended daily gram amounts^44^. These countries, according to WHO^10^, are suffering from diet-related problems, or NCDs and CVDs, because of the high intake of fat/oil, sugar, and salt. These are the culprits behind many diet-related problems. Though our study does not measure the gram intake for the households, the recall for the 48-h period indicates there is a higher chance the meals may or may not exceed the required intakes if this pattern of consumption continues as shown from their recalls. We can only presume from the results that their health may result from some level of nutritional deficiency, seeing that some food groups are either lacking or consumed more in their diet.

### Household dietary pattern: accessibility and availability

The dietary pattern during the 48-h recall survey revealed that several food groups were not consumed. For example, consider insects, non-alcohol beverages, eggs, alcoholic beverages, and dairy products. However, this does not imply that households never consumed these food groups since, in the last 12 months’ recall, these food groups were mentioned, indicating they have access to these foods but consume them moderately or infrequently (Fig. 4a), as not all family members consume them. The moderate or infrequent consumption of eggs, alcoholic beverages, and dairy products could be due to the economic status of some households since these products are often purchased from shops and thus may be unaffordable when compared to their income. Others, such as non-alcohol drinks and insects, depend on the seasons and their availability, respectively. Apart from seafood (mostly fish) and livestock (mostly chicken and pig), the consumption of insects is an additional source of good protein; however, not all households are consuming or have preferences for these products. However, the pattern of food groups-and-sources diversity shows a high intake of condiments, oils/fats, carbohydrates, protein from animals, vegetables, and sugary products for energy. Fruits are not recalled as some of the fruit varieties and nuts are seasonal and thus can only be accessed during their season of availability. Additionally, some of the food accessibility problems faced are during events where the harvest was not matured or damaged by extreme weather events (example, cyclones, extended hot and rainy days), thus, households often bought food from the markets. Legumes and nuts/seeds were mostly sourced from the bush and gardens. Grain/cereal, sugary goods, and tinned meat were obtained from the small shops, while fresh meats were obtained from the sea (example. fish, octopus, crabs, and squid) and livestock farms (example, chicken and pork). 

Based on Figs. 3, 4b, and 6, there is a similar trend where the majority of the food items are gathered from the sea and shops/stores. These are common food sources seen among the studied population. While the 48-h recall does not show households obtain food from others (relatives) or kustom/community/church events, the 12-monthly recall revealed that it is a usual practice among the rural population, which is also reported by^13,33^.

This indicated that households can access food on their own from multiple sources. We were able to identify that the following food groups—eggs, insects, dairy, savory, non-alcohol beverages, alcoholic beverages, pre-cooked foods, and sweets—foods/snacks—were the least recalled compared to other food items on a daily, two-to-three times a week, weekly, infrequently, and fortnightly basis in the past 12 months. However, from both recalls, most of the pre-cooked foods are mostly take-out, baked, or barbequed foods sold in the market (Tables 5 and 6). Based on the pattern seen in the past 12 months and compared to their 48-h recall, the tendency to improve their diet to sustain a well-balanced diet still needs improvement.

**Table 5:** Main food groups and the subdivided groups for 2018.

Grain, white roots and tubers, and plankton	%	Pre-cooked foods	**%**	Other fruit	**%**
Giant taro	40.20	***(*Optional beverages/food)**		Water melon	12.40
Banana	51.50	Fish and chips	4.10	Pineapple	43.30
Taro	94.80	Baked fish	4.10	Cocoa	9.30
Sweet potato	93.80	Take away	1.00	Guava	40.20
Yam	52.60	BBQ fish	1.00	Orange	13.40
Chinse taro	56.70	Pop corn	17.50	Lemon	4.10
Cassava	70.10	Fish roll	2.10	Five corner	16.50
Pana	25.80	**Nuts and seeds**		Pomelo	4.10
Giant swamp taro	4.10	Ngali nut	91.80	Breadfruit	20.60
Rice	68.00	Cut nut	45.40	Sweet banana	21.60
Bun/scone/bread	28.90	Peanut	19.60	Mango	8.20
Biscuit	11.30	Terminalia kaernbachii	6.20	Malay apple	12.40
Ring cake/ball cake	39.20	Betelnut	8.20	Soursop	4.10
Banana chip	14.40	**Dairy products**		Golden apple	3.10
Cassava chips	2.10	Butter	3.10	Coconut juice	26.80
Pan-cake	12.40	**Oils and fats**		Pineapple juice	5.20
Bean cake	6.20	Coconut cream	97.9	Orange juice	2.10
Potato chips	5.20	Cooking oil (soya)	60.80	Melon juice	2.10
Taro pudding	7.20	Cooking oil (chilly)	5.2	**Meat, poultry, and seafood**	
Cassava pudding	9.30	**Sugary products**		Pig	73.20
**Other vitamin A-rich fruits, vegetables and roots and tubers**		Sugarcane	11.30	Chicken	40.20
Pumpkin	20.60	Pop drink	32.00	Duck	2.10
Pawpaw	48.50	Ice block	26.80	Fish	100.00
Mango juice	1.00	Lemon tea	30.90	Mussels/.shells	71.10
Pawpaw juice	2.10	Soft drinks	19.60	Seaweeds	15.50
**Other vegetables**		Lemonade	4.10	Squid	13.40
Corn	20.60	Milo	3.10	Crabs	7.20
Eggplant	41.20	Tea products	78.40	Octopus	6.20
Cucumber	23.70	Coffee products	29.90	Lobster	5.20
Pepper	60.80	Coffee-mix	27.80	Prawn	1.00
Spring onion	57.70	Lolly	32.00	Lucas	29.90
Tomato	78.40	Gum	5.2	Sego grubs	36.10
Bean	93.80	Cream biscuit	12.40	**Egg**	
**Condiment**		Twisties	3.10	Chicken egg	23.70
Salt	96.90	Cake	7.20	**Dark green leafy vegetables**	
Curry powder	19.60	Ice cream	2.10	Dark green veggies	80.40
Black source	9.30	Chocolate	1.00		
Chilly	25.80	Solbrew/beer	17.50		
Ginger	30.90	**Home-made alcohol**	3.10		
Onion	6.20	Hot staff	1.00		
Garlic	10.30	Wine	1.00		
Spices	10.30	Sugarcane juice	1.00		
Corn flour	2.10				
Oyster source	1.00				
Sugar	14.40

**Table 6:** Food items that makes up the food groups: Produced or consumed in 2018.

Food items	Freq	%	Food items	Freq	%	Food items	Freq	%
**Vegetables/crops**			**Fruits**			**Fresh meats**		
Giant taro	39	40.20	Water melon	12	12.40	Pig	71	73.20
Banana	50	51.50	Pineapple	42	43.30	Chicken	39	40.20
Dark green veggies	78	80.40	Pawpaw	47	48.50	Duck	2	2.10
Taro	92	94.80	Cocoa	9	9.30	**Egg**		
Sweet potato	91	93.80	Guava	39	40.20	Chicken egg	23	23.70
Pumpkin	20	20.60	Orange	13	13.40	**Sea foods**		
Yam	51	52.60	Lemon	4	4.10	Fish	97	100
Chinse taro	55	56.70	Five corner	16	16.50	Muscles/.shells	69	71.10
Cassava	68	70.10	Pomelo	4	4.10	Seaweeds	15	15.50
Tomato	76	78.40	Breadfruit	20	20.60	Squid	13	13.40
Pepper	59	60.80	Sweet banana	21	21.60	Crabs	7	7.20
Cucumber	23	23.70	Mango	8	8.20	Octopus	6	6.20
Corn	20	20.60	Malay apple	12	12.40	Lobster	5	5.20
Spring onion	56	57.70	Soursop	4	4.10	Prawn	1	1.0
Eggplant	40	41.20	Golden apple	3	3.10	**Fats/oil**		
Sugarcane	11	11.30	**Insects**			Coconut cream	95	97.90
Pana	25	25.80	Lucas	29	29.90	Cooking oil (soya)	64	65.97
Giant swamp taro	4	4.10	Sego grubs	35	36.10	**Sugar sweet beverages**		
**Non-alcohol beverages**			**Cereal/grains**			Tea-lipton	59	60.80
Coconut juice	26	26.80	Rice	66	68.00	Pop drink	30	30.90
Pineapple juice	5	5.20	Bun/scone/bread	28	28.90	Ice block	26	26.80
Sugarcane juice	1	1.00	Biscuit	11	11.30	Milk tea	27	27.80
Pawpaw juice	2	2.10	**Savory**			Lemon tea	30	30.90
Orange juice	2	2.10	Ring cake/ball cake	38	39.20	Tea master	10	10.30
Melon juice	2	2.10	Banana chip	14	14.40	Soft drinks	19	19.60
Mango juice	1	1.00	Cassava chips	2	2.10	Lemonade	4	4.10
**Alcohol beverage**			Pop corn	17	17.50	Coffee-mix	27	27.80
Solbrew/beer	17	17.50	Pan-cake	12	12.40	Milo	3	3.10
Home-made alcohol	3	3.10	Fish roll	2	2.10			
Hot staff	1	1.00	Bean cake	6	6.20	**Legume**		
Wine	1	1.00	Potato chips	5	5.20	Beans	91	93.80
**Pre-cooked foods**			**Condiments**			**Sweets-foods/snacks**		
Taro pudding	7	7.20	Salt	94	96.90	Lollies	31	32.00
Cassava pudding	9	9.30	Curry powder	19	19.60	Gum	5	5.20
Fish and chips	4	4.10	Black source	9	9.30	Cream biscuit	12	12.40
Baked fish	4	4.10	Chilly	25	25.80	Twisties	3	3.10
Take away	1	1.00	Ginger	30	30.90	Cake	7	7.20
BBQ fish	1	1.0	Onion	6	6.20	Sugar	14	14.40
**Nuts/seeds** c			Garli	10	10.30	Ice cream	2	2.10
Ngali nut	89	91.80	Spices	10	10.30	Chocolate	1	1.00
Cut nut	44	45.4	Corn flour	2	2.10	**Dairy products**		
Peanut	19	19.600	Oyster source	1	1.00	Butter	3	3.10
Terminalia kaernbachii	6	6.200						
Betelnut	8	8.200						

The study has revealed that the average HDDS for the communities was five, while independently the HDDS for zone two households was six, and zone one maintained the average of five groups. The differences in community consumption may be associated with the locations. For instance, the high HDDS in zone two could be due to the location and accessibility of other food groups in the shops or markets. In addition, zone two is a mini-town, thus having more advantages in terms of access to shops and markets compared to zone one, where access to shops and markets is minimal. There is also high purchase power in zone two compared to zone one. However, while the study revealed higher purchasing power for zone two households than zone one households, it is lower than the purchasing power of the Auki population (the main town area in Malaita Island), whose food items were primarily obtained from the Auki market and the shops, as reported in a previous study^45^.

Moreover, several studies^4,46,47^ have reported that there is a shift from a diet high in root vegetables, coconuts, and fresh fish, bread, rice, tinned fish, and processed foods that are high in sugar and salt. This has resulted in a rise in NCDs. Looking at the dietary pattern of the studied communities, the probability of nutritional deficiency among households in the two zones will also increase. According to Troubat et al.^17^, a lack of nutritional diet has resulted in 30.00% of children under the age of five being stunted and 41.00% anemic.

It was intriguing to see that, from the average HDDS, 90.70% of households consume more than five food groups, which is above the WHO recommended consumption. However, if we are to look at the 13 food groups, all the households fell below the average, or 6.5 or 7 food groups, within the 48-h. This only indicates the household’s accessibility to various food groups. Nevertheless, looking at the five food groups consumed in the context of the local community’s studies, these are considered their main staple foods (grain, white roots, and tubers; plantains; oils/fats; condiments; dark green leafy vegetables; and meat/poultry/seafood. From the analysis, this indicated a good intake and accessibility of micronutrients from the food groups. However, only 9.10% are below the required food group intake.

This implies that a large percentage of households are more likely to achieve better micronutrient adequacy^48^, while only a small percentage are likely to experience nutrition inadequacy, an inability to acquire food, and food insecurity. This is supported by Martin-Prevel et al.^48^, justifying that, although their studies use a different number of food groups, households that consume more than five food groups are more likely to achieve better micronutrient adequacy. Therefore, although our study used 13 food groups, compared to previous studies^21,49^, it supported our results on the lack of DD affecting rural communities’ diets, although studies by^2,3,50^ reported high Individual Dietary Diversity Score (IDDS) ranging from five to seven point three. Our mean HDDS was similar to a study done in Papua New Guinea (PNG) by^33^ and in the Solomon Islands^3^. However, the HDDS contrast for this study was higher than another study in PNG by approximately three^51^ ˂four in Kiribati^34^, four-point seventeen in Malawi^52^, four-point nine in Bangladesh^32^ but lower than seven point eight in Fiji^36^, six point five and seven-point three in urban Solomon Islands^2,50^. However, all the studies are on urban populations except PNG^51^, Malawi^52^, and Bangladesh^32^, which suggested that the rural DDS for zone two and zone one are still high in terms of the rural population and the type of individual food groups consumed (HDDS). Although the total number of food groups used per study was different, they all consumed the recommended food group. Furthermore^53^, stated that one should keep in mind that the quality and quantity of foods in the diet may fluctuate depending on the production cycle or the source from which the foods are obtained. This can also influence farmers’ income and accessibility.

The dietary pattern for the two zones showed good accessibility to and availability of food items, which they were able to provide for their households. However, income, family size, and the number of dependents is also contributing factors to their diet. These factors can determine how much a household can consume, plus the dietary requirements of its members. For example, Kiribati's diet shows high accessibility to store-bought foods, mostly consisting of cereal/grain staples, showing a high dependence on processed food compared to our study, where the typical diet consisted mostly of garden staples followed by store-bought staples consumed on certain occasions. The differences may be because of income, accessibility to store foods, and the background of the locations in terms of gardening (atolls and volcanic islands).

Furthermore, our HDDS results reveal that 55.00% of the households have low to medium HDDS, while 44.80% have a higher HDDS (Table 3). Compared to studies in Fiji^36^ and Kiribati^34^ 85.00% and 97.30% have low to medium HDDS, respectively. This means that compared to the current study, this was a typical trend among the rural population in most least-developed and developing nations. This could be due to a lack of appropriate information to maintain or attain an appropriate diet for households to satisfy their hunger and improve their health.

### Correlation between socio-demography and diet

Attempting to examine the diet pattern in relation to demographics, we discovered a very low correlation of 0.174* between income and total meals. However, individually, zone one does show a positive relationship between income and day two total meals (0.263*) and family size (0.275*), meaning that income does influence their eating habits and the variety of food groups they consume. On the other hand, zone two has a closer relationship with income with day one meal ingredients (0.240*), day one total food groups (0.279*), and day two meal ingredients (0.321*). This suggests that they have greater accessibility to other food groups, which may be influenced by their location and employment status. However, according to the combined data (Table 4), income has minimal influence on the overall household dietary pattern. This is reasonable given that most (if not all) households in rural communities rely heavily on gardens, other than stores/shops, as their daily source of food. We also observe a strong relationship between total dependents (0.236**), family size (0.215*), and total meals. This means households with a large number of dependents and family size will have a high demand for daily meals. With limited availability of food to cater to the large family’s meals, some members of the family have reported skipping meals and prioritizing only certain members of their family during meal times. As one participant stated, “*Yes. Especially when the extended families visit and there is not enough food, I skip meals and give it to the grandchildren*” (zone two participant, age 37).

It was also discovered from the results there was a strong correlation of 0.504** between HDDS and total meals (48-h), implying that the overall number of food groups consumed within a household during the two-day recall had a significant relationship with the total number of meals (average three) with an average of five food groups consumed. It indicates that the households are food-secured one way or the other considering the level of accessibility and availability of food and the sources.

### Food and nutritional security within a household

Nonetheless, due to the variations in the food groups consumed by households, not all households consume a balanced meal. Because condiments were part of the food groups, they made up the fifth food group consumed. However, if condiments were removed due to their small quantity in most dishes, there is a high probability that many families would consume only four food groups, the baseline for the WHO recommended consumption intake. Several studies^48,54,55^, suggested that, even if assessing servings or an adequate amount is impossible, it is advisable to try to exclude very small quantities during the assessment. This is because the relationship between food group diversity and micronutrient adequacy becomes stronger when very small amounts of a food group are excluded from the count. However, our study does not measure the micronutrients; thus, the total food groups consumed show good economic accessibility by the households. Additionally, the majority of locally grown food consumed in our study were common crops grown and consumed around the Pacific Island Countries (PICs). According to Chandrasekara^56^, crops contain one or more bioactive compounds or nutrients that are necessary for good health. However, the nutrients may vary in the diet, which may mean that most households will still suffer from nutritional deficiency, likely leading to malnutrition. This could also be the reason for the studied population's daily consumption of high roots and tuber crops.

Protein is one important requirement in a diet and can be obtained from both plants and animals. One of the plant-based proteins with a high weight (Table 7) is pulse; however, it was shown to be consumed less. It was also evident that within the typical food groups consumed, households consumed at least one source of protein from animal products, nuts/seeds (depending on the season), fresh plant-based protein products, fruits, and garden or store staples contributing to their dietary protein, as^48^ also found. For example, the observed food intake pattern reveals that all households consume a highly starchy diet containing grains (floury products with rice), fresh white roots and tubers, and plantains, mostly sweet potato and taro, with 35.70%–95.90% of energy coming from protein obtained from sugary products, oils/fats, raw meat, which includes tinned tuna (fresh and processed). Nevertheless, all the food groups that make up the meals are consumed with fresh vegetables (94.80%).

**Table 7:** Allocated weight given to the different food groups.

Food Items	Food group	weight
1: Bread, noodles, biscuit, flour balls or foods made from maize, rice, and wheat. Yam, cassava, white sweet potato, Chinese taro, taro (*C.esculenta*), banana, breadfruit, sago	Cereals/tubers	2
**2:** Beans, peas, peanut	Pulses	3
3: Green leafy vegetables and other vegetables	Vegetables	1
4: Fruits and other fruits	Fruits	1
**5:** Beef, poultry, pork, eggs, fish, and other small protein	Meat/fish	4
6: Milk and other dairy	Milk	4
7: Sugar and sugary products	Sugar	0.5
8: Oil, fats and coconut cream	Oil	0.5
9: Condiments (food additives, tea products)	Condiments	0
10: Tinned fish/meat food	Tinned fish/meat	2

In terms of food preparations, most of the meals were either baked or boiled with coconut milk, which resulted in the high percentage of oils/fats for the 48-h diet recall and record for the past 12 months. This was accounted for as a typical dietary pattern for the households. In the rural Solomon Islands, Albert^21^ found a similar trend, reporting that daily meals frequently consist of fish, sweet potatoes (and/or rice), and slippery cabbage (a green leafy vegetable) cooked in coconut milk or baked. Horsey^2^ in terms of the percentage of food items consumed in their study also supported our findings.

However, according to FAO and SPC^57^, the food intake behavior for the Solomon Islands indicated that the overall national energy from diet consumption was around 2640 kcal per day, with some differences at the subnational level, implying that the average cost to acquire 1000 kcal would range between SBD$ 4.9 (the lowest) and SBD$ 7.5 (the highest). As per our study results, the majority of households consume low-to-moderate levels of food groups, which we suggested would fall between the findings by^17^. This indicated that our studied household's dietary habits are directly connected to the size of their household, income, and ability to acquire additional food products. Most households' vitamin A-rich requirements are met by consuming dark green leafy vegetables, which are also high in folate and several other micronutrients^58^, with only 6.10% coming from other vitamin-A-rich fruits and vegetables like pumpkin, ripe pawpaw, and mango, and 15.30% coming from other fruits. Unsaturated fatty acids, vegetable protein, fiber, minerals, tocopherols, phytosterols, and phenolic substances are also abundant in nuts and seeds. They all have high fat content, which may provide health benefits to households^59–61^. However, they are less commonly consumed due to their seasonal patterns.

Thus, it was evident from the 12 months of food group-and-source recalls and 48-h HDDS that households had a good variety of food sources and several meals per day, despite a small percentage of households being deprived of consuming more than five food groups within 48-h. Although^21^ malnutrition research in the Solomon Islands demonstrated their population is food secure. Our data suggest that the majority of the households managed to obtain five acceptable food groups, but the majority fell below the average of six-point five food groups, meaning their accessibility and availability to a variety of food is minimal. However, since the current study does not go into detail in measuring the nutrient content and gram or serving consumed per individual, the probability of the household members suffering from or facing nutrient deficiency is still large. It still shows that the households still have good food accessibility and are still food secure in a rural context, as shown by the average food groups consumed and accessed.

## Conclusion

Thus, household food accessibility patterns in zones one and two have not changed significantly over the course of one year based on the 12 months of comparing their food groups-and-sources recalls. They have demonstrated sufficient food group intake, meeting the WHO’ average recommendations based on their HDDS. However, certain households suffer a significant risk of food insecurity because they fail to meet the necessary nutritional standards in their daily intake based on the data obtained (HDDS). This was because some food groups are only available seasonally or because disasters such as cyclone damaging their food products. Households do have specific purchasing power, whether on a daily or irregular basis, proving their economic access to particular food groups. Despite the consumption of five food groups and high HDDS, when compared to other remote regions, these two rural zones are still not close to achieving a well-balanced and adequate diet, resulting in nutrient insufficiency. However, because the present study does not go into detail in evaluating nutritional content and the gram or portions consumed, the likelihood of household members suffering or facing nutrient deficiencies remains high. Nonetheless, the overall HDDS is good; however, further nutritional studies to investigate the macronutrients and micronutrients will be of great assistance to their health.

## Data Availability

The data supporting this study is all represented in the manuscript.
